# Less-Invasive Hemodynamic and Tissue Perfusion Monitoring in Sepsis and Septic Shock: A Narrative Review

**DOI:** 10.3390/jcm15052061

**Published:** 2026-03-08

**Authors:** Marialaura Scarcella, Paolo Formenti, Gian Marco Petroni, Riccardo Monti, Edoardo De Robertis

**Affiliations:** 1 Anesthesia and Intensive care Department, Perugia University, 06121 Perugia, Italy; 2 Critical Care and Anesthesiology Department, Ospedale Bassini, ASST Nord Milano, Cinisello Balsamo, 20095 Milan, Italy; 3 Anesthesia and Intensive care Unit, Azienda ospedaliera Santa Maria of Terni, 05100 Terni, Italy; 4 Anhestesiology and Operating Room Department, Azienda ospedaliera Santa Maria di Terni, 05100 Terni, Italy

**Keywords:** sepsis, septic shock, hemodynamic monitoring, tissue perfusion, less-invasive monitoring, critical care

## Abstract

Sepsis and septic shock remain major causes of morbidity and mortality in critically ill patients. Hemodynamic management is a cornerstone of treatment, yet the optimal monitoring strategy to guide resuscitation is still debated. The progressive decline in the use of invasive techniques, such as pulmonary artery catheterization, has favored the development of less-invasive and non-invasive monitoring approaches. Recent technologies allow continuous assessment of cardiovascular function through arterial waveform analysis, non-invasive blood pressure monitoring, and predictive algorithms, while increasing attention has been directed toward the evaluation of tissue perfusion and oxygenation. This reflects the recognition that normalization of macrocirculatory variables does not necessarily ensure adequate microcirculatory perfusion in sepsis. This narrative review summarizes current evidence on less-invasive hemodynamic and tissue perfusion monitoring in sepsis and septic shock, discussing their physiological rationale and potential role within contemporary, multimodal resuscitation strategies.

## 1. Introduction

Sepsis, defined as life-threatening organ dysfunction caused by a dysregulated host response to infection, and its most severe form, septic shock, constitute a major global health priority [1]. In 2017 alone, an estimated 49 million cases of sepsis and 11 million sepsis-related deaths occurred worldwide [2]. The pathophysiology of septic shock is complex, involving profound macro- and microcirculatory disturbances. These include vasodilation, myocardial dysfunction, and a compromised microcirculatory flow distribution, which collectively impair end-organ perfusion and lead to multiple organ failure [3].

Early and appropriate hemodynamic resuscitation represents a cornerstone of sepsis management, alongside prompt source control and antimicrobial therapy [4]. Current Surviving Sepsis Campaign guidelines emphasize the urgency of initiating resuscitative measures in patients with sepsis-induced hypoperfusion or septic shock [5]. Historically, invasive monitoring with the pulmonary artery catheter (PAC) played a central role in guiding therapy, providing measurements such as central venous pressure and cardiac output [6]. However, its routine use has progressively declined, largely due to concerns related to invasiveness and the absence of consistent evidence demonstrating improved clinical outcomes [7,8].

These limitations have driven the development and clinical adoption of a new generation of less-invasive and non-invasive hemodynamic monitoring technologies. Such systems aim to deliver continuous, real-time information on cardiovascular function while reducing procedural risks. Integrated platforms, including advanced monitoring systems capable of combining pressure, flow, and tissue perfusion data, reflect a broader shift toward individualized and physiology-based resuscitation strategies [9]. This evolution mirrors the transition from rigid, protocol-driven management toward a more adaptive approach, in which hemodynamic targets are interpreted in the context of each patient’s underlying pathophysiology [10].

In this review, we provide an overview of contemporary less-invasive and non-invasive hemodynamic monitoring technologies and discuss their potential role in the management of sepsis and septic shock. Particular attention is given to arterial waveform analysis, predictive algorithms for hypotension, and techniques for assessing regional tissue perfusion. Finally, we examine how these tools may be integrated into modern resuscitation strategies, informed by recent clinical trials and international guidelines, with the aim of supporting more personalized hemodynamic management in critically ill patients.

## 2. The Pathophysiological Basis for Advanced Monitoring in Sepsis

The circulatory failure associated with sepsis is multifaceted and involves disturbances at both the macro- and microcirculatory levels. At the macrocirculatory level, septic shock typically presents hypotension due to arterial and venous vasodilation, often accompanied by sepsis-induced myocardial dysfunction [11]. Traditionally, hemodynamic resuscitation strategies have focused on correcting these systemic alterations, targeting variables such as mean arterial pressure (MAP) and cardiac output (CO), based on the assumption of a direct relationship between global blood flow and tissue oxygen delivery [3]. However, a feature of sepsis is the frequent dissociation between macrocirculatory stabilization and microcirculatory perfusion, a phenomenon described as “hemodynamic incoherence” [12]. In this setting, normalization of systemic hemodynamic parameters does not necessarily translate into improved tissue oxygenation. Sepsis-related microcirculatory dysfunction is characterized by heterogeneous capillary blood flow, endothelial activation, glycocalyx degradation, increased capillary permeability, and altered flow distribution, all of which contribute to regional hypoxia and organ dysfunction [13,14].

Mechanistically, hemodynamic incoherence in sepsis arises from a combination of altered vascular regulation and impaired microvascular architecture. Endothelial activation and loss of glycocalyx integrity promote capillary leakage, interstitial edema, and a reduction in effective perfused capillary density, while inflammation-driven changes in rheology and microthrombosis contribute to marked flow heterogeneity and functional shunting [15]. In this context, increases in global blood flow or vasopressor-driven restoration of MAP may preferentially recruit already well-perfused regions, leaving hypoperfused territories unchanged. Clinically, this dissociation is associated with persistent regional hypoxia, impaired oxygen extraction, and ongoing organ dysfunction despite apparently “adequate” macrocirculatory endpoints [16]. Importantly, these concepts have practical implications: macro-hemodynamic targets should be interpreted as necessary but not sufficient, and the persistence of hypoperfusion signs should prompt a structured reassessment of the oxygen delivery chain. Rather than escalating fluids or vasopressors reflexively, an individualized strategy should integrate systemic variables with peripheral perfusion markers and, when available, microcirculatory surrogates to better match the choice and dose of fluids, vasopressors, and inotropes to the predominant phenotype (vasoplegia, myocardial dysfunction, or impaired microvascular recruitment).

In addition, cellular and metabolic alterations further complicate the interpretation of conventional hemodynamic markers. Mitochondrial dysfunction and impaired oxygen utilization may lead to so-called “cytopathic hypoxia”, a condition in which venous oxygen saturation can remain normal or even elevated despite ongoing tissue hypoxia [17]. As a result, dependence on global hemodynamic variables alone may provide an incomplete or misleading assessment of the adequacy of resuscitation [18].

Against this pathophysiological background, the management of shock has traditionally relied on invasive monitoring to guide therapy [19]. The PAC was once considered the gold standard, as it provided comprehensive information on cardiac filling pressures, cardiac output, and mixed venous oxygen saturation (SvO_2_) [20]. However, its routine use has progressively declined after large randomized controlled trials, including the PAC-MAN study [21], failed to demonstrate a significant reduction in hospital mortality compared with standard care, leading to a less clearly defined role for pulmonary artery catheterization in the era of newer and less-invasive monitoring techniques. In addition to the uncertainty regarding its impact on mortality and length of stay, the use of a pulmonary artery catheter is associated with non-negligible risks. Procedure-related complications include vascular injury, arrhythmias during catheter advancement, catheter-related bloodstream infections, thrombosis, and, more rarely, pulmonary artery rupture, a potentially fatal event [22]. Misinterpretation of complex hemodynamic variables may also lead to inappropriate therapeutic decisions [23]. Although the incidence of severe complications is relatively low in experienced centers, the invasive nature of the technique and its safety profile have contributed to the progressive shift toward less-invasive or non-invasive monitoring strategies, particularly when advanced hemodynamic information can be reasonably approximated using alternative tools.

This shift has led to the widespread adoption of minimally invasive and non-invasive hemodynamic monitoring systems that offer a more favorable risk–benefit profile while still providing continuous and clinically relevant information [24]. These technologies mainly rely on arterial pressure waveform analysis or non-invasive methods, such as finger cuff plethysmography, to derive key hemodynamic parameters [25].

## 3. Modern Less-Invasive and Non-Invasive Monitoring Platforms

Over the last decade, several less-invasive and non-invasive systems have been developed with the aim of integrating data derived from arterial pressure waveform analysis, continuous blood pressure monitoring, and, in selected cases, indices of tissue perfusion or oxygenation within a single monitoring environment.

Rather than relying on a single parameter, these platforms are designed to simultaneously display pressure-, flow-, and perfusion-related variables, facilitating a more comprehensive interpretation of the patient’s circulatory status at the bedside [26]. Different technological solutions are currently available, with varying degrees of integration, ranging from modular multiparametric monitors to more consolidated platforms that incorporate advanced signal processing and clinical decision-support tools. Reviews comparing available technologies consistently describe different methodological families, including pulse contour and pulse wave analysis, thoracic bioimpedance, and bioreactance, each based on distinct physical and physiological principles [27]. The accuracy and reliability of these approaches may vary according to vascular tone, heart rhythm, mechanical ventilation, and signal quality, with relevant limitations in patients with profound vasodilation or circulatory instability, such as those with septic shock [28].

Among fully non-invasive methods, techniques based on thoracic electrical bioimpedance and bioreactance have been developed to estimate stroke volume and cardiac output by analyzing changes in thoracic electrical properties during the cardiac cycle [29,30]. Some authors showed that their performance may be affected by thoracic fluid shifts, lung pathology, and changes in vascular resistance, limiting their applicability in critically ill patients. Table 1 and Table 2 summarized principles, variables, limitations, and applicability of less-invasive and non-invasive hemodynamic monitoring.

### 3.1. Arterial Waveform Analysis: Principles, Clinical Applications, and Limitations

Over the last decade, arterial waveform analysis has remained one of the most extensively studied minimally invasive approaches for continuous hemodynamic monitoring in critically ill patients. While early methodological work established the theoretical relationship between arterial pressure, arterial compliance, and systemic vascular resistance, more recent studies have consistently emphasized that pulse contour–based systems are primarily reliable for tracking directional changes rather than providing accurate absolute cardiac output values across heterogeneous ICU populations. In a detailed contemporary review, Grensemann [53] highlighted how alterations in vascular tone and arterial impedance represent major sources of error, particularly in critically ill and vasoplegic patients.

Building on this framework, several investigations over the past years have focused on dynamic indices derived from arterial waveform analysis, including pulse pressure variation (PPV), systolic pressure variation (SPV), and stroke volume variation (SVV), as functional markers of preload responsiveness [33]. The clinical relevance of respiratory-induced arterial pressure changes was further established by Michard et al. [49], who demonstrated that pulse pressure variation accurately predicts fluid responsiveness in septic patients receiving controlled mechanical ventilation.

Over the last two decades, Teboul and colleagues have contributed substantially to defining the physiological prerequisites and clinical limitations of dynamic indices. In a pivotal review, they summarized the conditions under which PPV and SVV can reliably predict fluid responsiveness, emphasizing the necessity of controlled mechanical ventilation, absence of spontaneous breathing activity, regular cardiac rhythm, and adequate tidal volume [54]. In a dedicated physiological analysis, Monnet et al. [55] showed that spontaneous breathing activity and irregular ventilatory patterns profoundly alter heart–lung interactions, thereby invalidating PPV and SVV. To overcome these limitations, they proposed the passive leg raising maneuver as a reversible “self-fluid challenge”, demonstrating its reliability across different ventilatory conditions [46]. Importantly, their later work also addressed the impact of lung-protective ventilation strategies. They demonstrated that low tidal volume ventilation markedly reduces the amplitude of respiratory-induced preload changes, limiting the diagnostic accuracy of PPV, and stressed that dynamic indices should be interpreted cautiously in modern ICU settings where protective ventilation is widely adopted [56].

The impact of ventilatory mode on the reliability of arterial waveform–derived indices has been further clarified by both earlier and more recent evidence. Zaniboni et al. [43] demonstrated that during partially assisted ventilation, irregular intrathoracic pressure swings markedly impair the predictive performance of PPV and SPV, independent of cardiac function. Subsequent observational studies and narrative reviews published in the last decade have confirmed that spontaneous breathing activity remains one of the most frequent reasons for dynamic index failure in daily ICU practice [24,30]. More recent work has specifically addressed the consequences of lung-protective ventilation strategies. Myatra et al. [52] showed that low tidal volume ventilation significantly reduces respiratory-induced arterial pressure variations, leading to a loss of diagnostic accuracy of PPV. They proposed the tidal volume challenge as a pragmatic bedside maneuver to transiently restore the physiological signal, demonstrating improved prediction of fluid responsiveness. This concept has since been reinforced by systematic reviews and meta-analyses published within the last few years, which confirmed that tidal volume challenge may enhance PPV feasibility, albeit only in selected, fully controlled ventilatory conditions [45,48,57].

Beyond ventilatory pattern and tidal volume, lung mechanics have emerged as a critical determinant of arterial waveform reliability. Teboul et al. [50] highlighted that in patients with ARDS or markedly reduced respiratory system compliance, the transmission of airway pressure to the intrathoracic compartment is attenuated, further limiting the performance of PPV and SVV even during controlled ventilation. Recent physiological studies and ICU-focused reviews have supported this concept, emphasizing that lung–chest wall mechanics should be considered when interpreting dynamic indices in critically ill patients [44,58].

Finally, the role of arterial waveform analysis in septic shock has been re-evaluated in light of recent evidence. Vasoplegia and rapid fluctuations in systemic vascular resistance profoundly alter the pressure–flow relationship underlying pulse contour analysis. In a recent systematic review and meta-analysis, Lamarche-Fontaneto et al. [48] reported wide limits of agreement and inconsistent clinical performance of arterial pressure–based cardiac output monitors in septic shock, concluding that their use should be primarily restricted to trend monitoring rather than absolute decision-making in this population.

### 3.2. Fully Non-Invasive Cardiac Output Monitoring: Bioimpedance and Bioreactance

Fully non-invasive techniques for cardiac output monitoring have been developed with the aim of avoiding arterial or central venous catheterization while still providing continuous information on stroke volume and cardiac output [59]. These methods are based on the analysis of thoracic electrical properties during the cardiac cycle, exploiting changes in impedance or phase shift generated by pulsatile aortic blood flow [60].

Thoracic electrical bioimpedance represents the earliest approach in this field. Initial validation studies demonstrated its feasibility for estimating cardiac output in controlled settings; however, subsequent investigations highlighted substantial variability in accuracy, particularly in critically ill patients [61]. Changes in thoracic fluid content, lung pathology, and electrode positioning were shown to significantly affect signal quality and measurement reliability, limiting the clinical applicability of bioimpedance in ICU populations [36]. These limitations raised early concerns regarding the applicability of bioimpedance in ICU populations.

To overcome some of these limitations, bioreactance was later introduced as a methodological refinement, based on the analysis of phase shifts rather than amplitude changes of the electrical signal [39]. Initial validation studies suggested improved signal stability and reduced sensitivity to noise compared with traditional bioimpedance. Squara and colleagues [62] evaluated bioreactance-based cardiac output monitoring in critically ill patients and reported acceptable agreement with reference techniques under stable hemodynamic conditions, while also documenting reduced accuracy during rapid changes in preload and vascular tone.

Subsequent comparative studies and reviews, however, provided a more cautious interpretation. Several authors reported that both bioimpedance- and bioreactance-based systems exhibit wide limits of agreement and clinically relevant percentage errors when compared with invasive reference methods, particularly in mechanically ventilated patients and in those with altered lung mechanics [30,63].

More recent evidence has further clarified the limitations of fully non-invasive cardiac output monitoring in unstable ICU populations. Studies focusing on septic shock consistently showed that profound vasodilation, rapid changes in systemic vascular resistance, and dynamic thoracic fluid shifts substantially impair the accuracy of impedance- and bioreactance-based measurements. A recent systematic review and meta-analysis specifically addressing septic shock reported poor agreement between non-invasive cardiac output monitors and reference techniques, concluding that these systems rarely provide clinically actionable information in this setting [48]. Similar conclusions were drawn in recent narrative and systematic reviews addressing non-invasive hemodynamic monitoring in critically ill patients, which emphasized that while bioimpedance and bioreactance may be useful for trend monitoring in selected, hemodynamically stable patients, their reliability markedly decreases in the presence of mechanical ventilation, significant lung pathology, or vasoplegic shock [64].

### 3.3. Tissue Perfusion and Oxygenation Monitoring: Microcirculatory Perspective

Microcirculation refers to the network of small vessels, including arterioles, capillaries, and venules, responsible for oxygen and nutrient delivery at the cellular level and for the removal of metabolic waste products [65]. In sepsis, microvascular dysfunction represents a key pathophysiological component and may persist even after apparent normalization of systemic hemodynamic variables. Monitoring tissue oxygenation therefore provides clinically relevant information that complements macrocirculatory parameters, as it reflects the balance between oxygen delivery and consumption at the regional level. Alterations in tissue oxygen saturation, particularly when persistent, have been associated with impaired oxygen extraction, mitochondrial dysfunction, and a higher risk of organ failure [66]. Consequently, integrating tissue oxygenation monitoring into a multimodal hemodynamic assessment may help identify patients with ongoing hypoperfusion despite adequate systemic targets.

Growing evidence over the last two decades has demonstrated that normalization of macrocirculatory variables does not necessarily ensure adequate tissue perfusion in critically ill patients. In sepsis and septic shock, microcirculatory alterations may persist despite restoration of arterial pressure and cardiac output, leading to ongoing tissue hypoxia and organ dysfunction. De Backer et al. [67] were among the first to demonstrate profound and heterogeneous microvascular alterations in septic patients using direct visualization techniques, establishing the concept of macro–microcirculatory dissociation. Subsequent experimental and clinical studies confirmed that microcirculatory dysfunction represents an independent determinant of outcome in sepsis. Sakr et al. [68] showed that the persistence of microvascular abnormalities over time, rather than their presence at a single time point, is associated with increased mortality. More recent evidence supports the prognostic relevance of simple bedside microcirculatory indicators in septic shock. In a retrospective cohort of septic shock patients, capillary refill time at admission, mottling score at 24 h, and perfusion index at 48 h were all independently associated with 28-day mortality, with the strongest predictive value observed for the persistence of a low perfusion index over time, reinforcing the concept that sustained microcirculatory dysfunction is closely linked to outcome [69].

To translate microcirculatory assessment into bedside practice, non-invasive techniques aimed at estimating tissue perfusion and oxygenation have been developed. Near-infrared spectroscopy (NIRS) emerged as one of the most extensively studied approaches, allowing continuous assessment of regional tissue oxygen saturation based on the differential absorption spectra of oxyhemoglobin and deoxyhemoglobin [70]. In particular, it allows to assess regional tissue oxygen saturation (StO_2_). Several clinical and physiological studies have shown that NIRS-derived cerebral (SctO_2_) and somatic (SmtO_2_) oxygenation reflect the balance between oxygen delivery and consumption and may detect regional hypoperfusion even when global hemodynamic variables appear preserved [40].

Clinical investigations subsequently explored the prognostic and physiological relevance of NIRS-derived variables in sepsis and shock. Studies conducted in septic patients demonstrated that low baseline tissue oxygen saturation and impaired recovery following ischemic challenges are associated with worse outcomes, even when systemic hemodynamic variables appear normalized [41,71]. More recent work has focused on the limitations of tissue oxygenation monitoring. Several authors emphasized that NIRS signals are influenced not only by local blood flow but also by tissue composition, venous-to-arterial blood volume ratio, and regional metabolic activity, complicating direct translation into therapeutic targets [72]. When integrated with systemic hemodynamic parameters such as arterial pressure, cardiac output, and hemoglobin concentration, tissue oximetry may help contextualize alterations in the oxygen delivery cascade, supporting clinical interpretation rather than serving as an independent resuscitation target [73].

#### Organ-Specific Perfusion Monitoring

In addition to global and peripheral perfusion markers, growing interest has focused on the direct assessment of organ-specific perfusion in septic shock. Systemic hemodynamic stabilization does not necessarily ensure adequate regional blood flow, and persistent organ hypoperfusion may occur despite apparently acceptable macrocirculatory parameters [74].

Among organ systems, the kidney has received particular attention due to the high incidence and prognostic impact of sepsis-associated acute kidney injury. Bedside Doppler ultrasonography allows non-invasive assessment of renal blood flow patterns and intrarenal vascular resistance through measurement of the renal resistive index (RRI) [75]. Although RRI is influenced by systemic hemodynamics, intra-abdominal pressure, and vascular compliance, several studies suggest that changes in RRI may reflect alterations in renal perfusion dynamics and correlate with the development or persistence of AKI in septic patients [76]. For instance, Oliveria et al. [77] showed how mean arterial pressure, lactate, age, and type of AKI might influence renal RI in critically ill patients. Importantly, interpretation requires integration with systemic variables, as RRI does not represent a direct measure of microcirculatory flow [78].

Contrast-enhanced ultrasound (CEUS) has emerged as a promising technique to evaluate renal cortical microperfusion at the bedside without exposure to nephrotoxic iodinated contrast agents [79]. Preliminary data indicate that CEUS-derived perfusion parameters may detect early microvascular alterations in sepsis and could potentially identify patients at risk of persistent renal dysfunction [79,80]. However, its use remains limited by availability, operator expertise, and the need for further validation in large prospective cohorts [81].

Beyond the kidney, assessment of organ perfusion has also been explored in the splanchnic and cerebral territories. Techniques such as transcranial Doppler, near-infrared spectroscopy applied to cerebral monitoring, and perfusion imaging with computed tomography have improved understanding of regional blood flow alterations in septic shock [82]. Nevertheless, the routine use of advanced imaging modalities is often constrained in unstable critically ill patients due to logistical challenges and the risks associated with transport and contrast exposure.

## 4. Multimodal Hemodynamic Monitoring: Integrating Microcirculation, Perfusion and Hemodynamic Coherence

Several primary clinical investigations have demonstrated that normalization of macrocirculatory variables does not necessarily translate into restoration of tissue perfusion [83]. A prospective study by Lara et al. [84] showed that prolonged capillary refill time was independently associated with mortality in septic patients with hyperlactatemia, even after adjustment for arterial pressure and vasopressor dose. In the randomized ANDROMEDA-SHOCK trial, Hernández et al. [85] compared a capillary refill time–targeted resuscitation strategy with lactate-guided therapy in septic shock. Although mortality reduction did not reach statistical significance, the study demonstrated that CRT-guided resuscitation resulted in different fluid and vasopressor trajectories, reinforcing the physiological relevance of peripheral perfusion assessment. Additional prospective evidence further supports the prognostic relevance of CRT in septic shock. In a 10-month prospective cohort study including 175 ICU patients with septic shock, CRT was measured at admission and 6 h after resuscitation [86]. While baseline CRT showed moderate discriminatory ability for 28-day mortality, CRT assessed at 6 h demonstrated markedly improved predictive performance.

Regarding mottling, Ait-Oufella et al. [87] demonstrated that the extent of mottling was strongly associated with mortality in septic shock patients. Importantly, subsequent analyses confirmed that persistence of mottling over time carries prognostic significance [88]. Metabolic flow surrogates have also been investigated. Ospina-Tascón et al. showed that a persistent elevation in the venous-to-arterial CO_2_ difference during early resuscitation identifies patients with inadequate blood flow despite normalization of arterial pressure [89]. This supports the interpretation of the CO_2_ gap as a marker of impaired flow–metabolism coupling rather than simply ventilation status.

Microcirculatory dissociation from macrocirculatory targets has been documented using direct microvascular imaging techniques. De Backer et al. [67] demonstrated that improvement in systemic hemodynamic variables does not necessarily correspond to restoration of sublingual microcirculatory flow in septic shock. Similar findings were reported by Trzeciak et al. [90], who showed that persistent microcirculatory abnormalities are associated with organ dysfunction and mortality.

Taken together, these original investigations indicate that macrocirculatory optimization alone may be insufficient to ensure effective tissue perfusion. Integration of systemic variables with peripheral perfusion assessment and metabolic flow markers may therefore improve physiological interpretation and guide resuscitation more precisely in septic shock.

## 5. Practical Implications: Translating Multimodal Monitoring into Bedside Decision-Making

**Figure 1:**
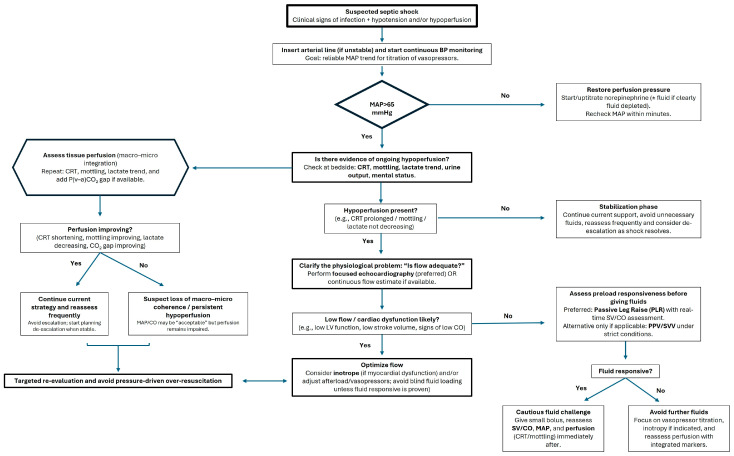
Practical flowchart for selecting and integrating hemodynamic monitoring in septic shock.

A practical approach to hemodynamic monitoring in septic shock should begin with clarification of the physiological question rather than the selection of a device (Figure 1). In the early phase of shock, the priority is restoration of perfusion pressure. Continuous invasive arterial pressure monitoring is recommended in unstable patients to ensure maintenance of a mean arterial pressure (MAP) of at least 65 mmHg, as supported by observational and interventional data indicating increased organ dysfunction and mortality with sustained hypotension [91,92]. However, achievement of MAP targets does not guarantee adequate tissue perfusion, and reliance on pressure alone may lead to false reassurance.

When signs of hypoperfusion persist despite adequate MAP (such as elevated lactate, prolonged CRT, or mottling) the clinical question shifts from pressure to flow. Functional hemodynamic assessment becomes relevant at this stage. The use of dynamic indices such as PPV and SVV has been shown to predict fluid responsiveness under strict physiological conditions, including controlled mechanical ventilation and sinus rhythm [49,93,94]. Importantly, these indices should not be applied indiscriminately, as their predictive performance deteriorates in spontaneous breathing, low tidal volume ventilation, or arrhythmias [33]. In such cases, PLR combined with real-time stroke volume measurement offers a reversible and reliable alternative for assessing preload responsiveness [45].

Before administering additional fluids, clinicians should determine whether increasing preloads are likely to augment cardiac output. Fluid administration in the absence of preload responsiveness may worsen venous congestion and tissue edema without improving perfusion. The FENICE study demonstrated substantial variability in fluid challenge practices across ICUs and highlighted the frequent absence of objective assessment of responsiveness [95], reinforcing the need for structured evaluation.

Even when MAP and cardiac output appear adequate, persistent abnormalities in peripheral perfusion should prompt reconsideration of tissue-level oxygen delivery. Prolonged CRT has been independently associated with mortality in septic shock [84], and the ANDROMEDA-SHOCK trial demonstrated that a resuscitation strategy targeting CRT leads to different physiological trajectories compared with lactate-guided therapy [85]. Additional mechanistic analyses confirmed that CRT-guided resuscitation is associated with earlier improvement in regional perfusion parameters [96]. Similarly, the presence and persistence of mottling have been shown to correlate with worse outcomes [87,88].

Integration of metabolic flow markers may further refine decision-making. The venous-to-arterial carbon dioxide difference has been shown to identify patients with inadequate flow relative to metabolic demand despite acceptable arterial pressure [97]. Combining lactate trends with CO_2_-derived indices may improve recognition of persistent hypoperfusion. Importantly, direct microcirculatory studies have demonstrated that restoration of systemic hemodynamic variables does not necessarily translate into recovery of microvascular flow underscoring the need for integrated interpretation rather than escalation of isolated targets [67]. Thus, multimodal monitoring should not be understood as the accumulation of devices, but as the structured integration of pressure, flow, perfusion, and metabolic signals to guide adaptive therapeutic decisions over time.

The algorithm illustrates a structured, physiology-oriented approach to hemodynamic evaluation in patients with suspected septic shock. The first step focuses on restoration of perfusion pressure (mean arterial pressure ≥ 65 mmHg), recognizing that adequate pressure is necessary but not sufficient to ensure tissue perfusion. Once systemic pressure targets are achieved, assessment of tissue perfusion (e.g., capillary refill time, mottling score, lactate kinetics, urine output) is performed to detect persistent hypoperfusion. In the presence of ongoing hypoperfusion, clarification of flow status is required, preferably through focused echocardiography or cardiac output estimation, followed by evaluation of preload responsiveness before fluid administration. Interventions (fluids, vasopressors, inotropes) are then tailored according to the predominant physiological disturbance. The final step emphasizes iterative reassessment and integration of macro- and microcirculatory variables to identify potential hemodynamic incoherence and to avoid pressure-driven over-resuscitation. The framework is intended as a conceptual model supporting individualized, multimodal decision-making rather than a prescriptive treatment guideline.

## 6. Conclusions

In sepsis and septic shock, accurate hemodynamic assessment cannot rely solely on systemic variables such as mean arterial pressure or cardiac output. Restoration of macrocirculatory targets does not necessarily guarantee adequate tissue perfusion, and persistent microcirculatory dysfunction may contribute to ongoing organ failure despite apparent hemodynamic stabilization. A multimodal monitoring strategy that integrates systemic hemodynamics, cardiac function assessment, and markers of peripheral or tissue perfusion provides a more comprehensive physiological evaluation. Less-invasive technologies may support this approach, particularly when used for trend analysis and repeated reassessment rather than as isolated decision-making tools. Future efforts should focus on defining standardized integration frameworks, identifying clinical phenotypes characterized by hemodynamic incoherence, and determining whether individualized, physiology-guided resuscitation strategies can translate into improved patient-centered outcomes.

## Figures and Tables

**Table 1:** Less-invasive and non-invasive hemodynamic monitoring: principles, variables, and limitations.

Monitoring Approach	Physiological Principle	Main Variables Provided	Clinical Strengths	Main Limitations
Arterial waveform analysis [31,32,33]	Analysis of arterial pressure waveform morphology and pulse contour	Cardiac output (trend), stroke volume, dynamic indices	Continuous monitoring; good trending ability	Accuracy affected by vascular tone, damping, arrhythmias
Dynamic indices (PPV, SVV, SPV) [31,34,35,36]	Heart–lung interactions during positive-pressure ventilation	Fluid responsiveness surrogates	Superior to static indices under ideal conditions	Unreliable with spontaneous breathing, arrhythmias, low tidal volume
Non-invasive continuous blood pressure monitoring [29,37]	Reconstruction of arterial waveform from peripheral signals	Beat-to-beat blood pressure, derived variables	Early detection of hypotension; no vascular access	Reduced accuracy in vasoplegia, hypothermia, peripheral edema
Thoracic bioimpedance/bioreactance [38,39]	Changes in thoracic electrical properties during cardiac cycle	Stroke volume, cardiac output (estimate)	Easy to use; completely non-invasive	Affected by lung pathology, fluid shifts, vascular resistance
Tissue oxygenation monitoring [12,40,41]	Near-infrared spectroscopy of regional hemoglobin oxygenation	Regional tissue oxygen saturation	Insight into microcirculatory perfusion	Limited outcome evidence; complex interpretation

**Table 2:** Applicability of less-invasive and non-invasive monitoring according to clinical conditions.

Clinical Condition	Potentially Useful Approaches	Approaches with Relevant Limitations	Key Considerations
Controlled mechanical Ventilation [42,43,44]	Dynamic indices, arterial waveform analysis	—	Regular rhythm and adequate tidal volume required
Spontaneous or assisted ventilation [45,46]	Echocardiography, passive leg raising	PPV/SPV/SVV	Irregular intrathoracic pressure swings invalidate dynamic indices
Septic shock with vasoplegia [47,48]	Multimodal approach, echocardiography	Arterial waveform absolute values	Vascular tone alters pressure–flow relationship
Cardiac arrhythmias [42,49]	Echocardiography	Dynamic indices, waveform analysis	Beat-to-beat variability limits reliability
ARDS/low lung compliance [33,44,50,51,52]	Echocardiography, tissue perfusion monitoring	Dynamic indices	Reduced transmission of airway pressure

## Data Availability

Data available upon reasonable request.

## Abbreviations

The following abbreviations are used in this manuscript:

CO	Cardiac Output
CRT	Capillary Refill Time
MAP	Mean Arterial Pressure
ICU	Intensive Care Unit
SvO_2_	mixed venous oxygen saturation
PAC	Pulmonary Artery Catheter
StO_2_	regional tissue oxygen saturation
SctO_2_	NIRS-derived cerebral oxygen saturation
PPV	Pulse Pressure Variation
P(v–a) CO_2_	Venous-to-Arterial Carbon Dioxide Partial Pressure Difference
SVV	Stroke Volume Variation
NIRS	Near-infrared spectroscopy
PLR	passive leg raising
